# Neglected Zoonoses and the Missing Opportunities for One Health Education: The Case of Cystic Echinococcosis among Surgically Operated Patients in Basrah, Southern Iraq

**DOI:** 10.3390/diseases7010004

**Published:** 2019-01-07

**Authors:** Mohanad F. Abdulhameed, Ian D. Robertson, Suzan A. Al-Azizz, Ihab Habib

**Affiliations:** 1 School of Veterinary Medicine, College of Science, Health, Engineering and Education, Murdoch University, Perth 6150, Australia; mohanad.abdulhameed@yahoo.com (M.F.A.); i.robertson@murdoch.edu.au (I.D.R.); 2 Veterinary Parasitology Division, College of Veterinary Medicine, University of Basrah, Basrah 61004, Iraq; suzanalazizz@yahoo.com; 3 China-Australia Joint Research and Training Center for Veterinary Epidemiology, Huazhong Agricultural University, Wuhan 430072, China; 4 High Institute of Public Health, Alexandria University, Alexandria 21516, Egypt

**Keywords:** cystic echinococcosis, hydatid disease, questionnaire, Iraq, One Health

## Abstract

Cystic echinococcosis (CE) is recognized as a neglected disease of public health significance throughout the world, particularly in low and middle-income countries. The objectives of this study were to describe the characteristics, attitudes, knowledge, and practices of some Basrah province residents diagnosed with CE. Using a questionnaire survey, we interviewed 50 surgically operated cases of CE from Basrah, south Iraq. The cases comprised of 31 females and 19 males, of which 74% originated from rural areas. The questionnaire contained 30 questions and focused on gathering the demographic characteristics of the patients and capturing their overall knowledge, attitudes, and practices toward CE. Approximately half of the participants reported slaughtering livestock at home for their families’ consumption, 78% indicated the presence of a large number of stray dogs roaming freely about their village, 86% reported that they never boiled water before drinking it, and 26% reported not washing vegetables before eating them. Although a large proportion of the participants (72%) had heard of hydatid disease before becoming sick, over half (57%) were not aware of how the disease can be transmitted from animals to humans. This study highlights a gap in One Health education efforts regarding CE in southern Iraq, with a lack of counselling of patients on how to prevent reinfection. An intensive One Health education program should be implemented in Basrah to reduce CE at the human–animal interface. Lack of awareness on zoonoses among medical professionals, who are supposed to disseminate advice on preventative measures to their patients, is a challenge to the public health system.

## 1. Introduction

Cystic echinococcosis (CE) is a neglected disease of public health significance in many low and middle-income countries [1]. The disease is caused by a tapeworm belonging to the genus *Echinococcus* which is transmitted between carnivores (dogs and wolves: The definitive hosts) and primarily livestock (the intermediate hosts), with humans accidentally acquiring the infection, usually through consuming food or water contaminated with eggs shed by the definitive host [2]. Once ingested by an intermediate host the eggs hatch in the small intestine releasing oncospheres, which penetrate the intestinal wall and circulate in the bloodstream to finally lodge in a vital organ where the cyst(s) slowly grow over several years [3]. Cysts in humans are predominantly found in the liver and the lungs [4,5].

The highest prevalence of CE in humans is typically found in sheep-raising rural communities, as sheep are considered the most important intermediate host. Also, the emergence of human echinococcosis may be attributed to a change in the local ecology and increasing urbanization resulting in greater exposure of people to infected dogs [6,7]. Several studies have highlighted the role of socio-demographic characteristics; including age, gender, occupation, and level of education as essential factors in the transmission of echinococcosis to humans [8,9,10].

In Iraq, CE is regarded as a major One Health concern [11]. The highest numbers of human cases have been reported in the provinces of central and southern Iraq including Basrah, Nasiriyah, and Muthana [12,13,14]. In recent research in Basrah, the annual clinical incidence of CE was estimated as 4.5 cases per 100,000 people based on hospitalization records reviewed between January 2005 to December 2015 [15]. In general, treatment of echinococcosis in humans is costly and complicated and needs a prolonged post-operative health care [16]. Hence, it is critical to ensure that people living in endemic communities receive appropriate health education on how to protect themselves from infection. Understanding the level of knowledge and awareness about the disease in a cohort of previously infected individuals is an important step toward identifying potential gaps that should be considered further when developing health promotion programs. In an endemic setting, such as in Iraq, failure to understand such gaps could hinder local control strategies and add burden to the public health system. The objectives of this study were to sketch in a descriptive way the characteristics, attitudes, knowledge, and practices of a cohort of patients who had undergone surgery as a result of CE in Basrah, Iraq.

## 2. Materials and Methods

### 2.1. Study Setting 

Basrah is the third largest province in Iraq and lies in the south of the country bordering Iran, Kuwait, and Saudi-Arabia. The human population in Basrah was estimated at 2.4 million in 2014, with 20.1% being rural [17]. The province contains almost 140,000 livestock (personal communication; obtained from the Veterinary Hospital of Basrah, Ministry of Agriculture based on the last census of livestock conducted in Basrah in 2015). In Basrah, there are six (five public and one private) hospitals that have the facilities and skilled surgeons to perform surgical operations on human cases of CE.

### 2.2. Cases Recruitment and Questionnaire Administration

This research was approved by the Human Ethics Review Committee of Murdoch University, Perth, Australia (Permission number: 2016/034). Official written approval to review hospital records and to contact patients was obtained from the Ministry of Health in Iraq and the Basrah Health Directorate. Of the six hospitals in Basrah that could provide treatment for CE, four approved the researcher to interview hospitalized CE patients after surgery or to contact patients who had been treated and discharged from 2014 to 2016. The hospitals were visited four times a week from May to July 2016, and throughout these site visits 19 current patients were recruited. A further 31 discharged patients were also approached and agreed to participate in the study. Hence, a total of fifty participants completed a questionnaire either at the hospital (face-to-face) for the 19 current patients or via telephone interview for the 31 discharged patients. Before administering the questionnaire, oral consents from all participants were secured. The parents/guardians of two female patients (4 and 10 years old) were interviewed on behalf of the children. The questionnaire instrument composed of 30 questions and focused on the demographic characteristics of the patients and their knowledge, attitudes, and practices related to CE. The questionnaire was written in English and translated into the Arabic language, by a native researcher from Iraq, and it was back-translated from Arabic to the English language to verify the clarity of translation. For all interviewed patients, the questionnaire was administered by the same researcher (the first author). The questionnaire instrument was based on a set of questions derived from a prior study where the instrument has been validated before, also in Basrah community [18]. A copy of the questionnaire can be obtained from the corresponding author upon request. 

### 2.3. Data Analysis

Data were entered into a spreadsheet (Excel, 2013, Microsoft Corporation, Washington, DC, United States) and descriptive analyses were undertaken using the Statistical Package for the Social Sciences version 20. Frequencies, percentages, and 95% confidence intervals (CI) for responses were calculated. Given the purposive selection of patients and their small number as a study cohort, it was decided to do only a descriptive analysis with no additional regression modelling; to avoid over-analysis and extrapolation of results that could be out of context.

## 3. Results

Sixty cysts had been surgically excised from the 50 participants. Around half of the cysts (51.7% (31/60)) were in the liver, and 28.3% (17/60) were in the lungs (Table 1). Most of the patients (82%) only had one organ affected, yet 16% had cysts in two organs, and one patient had three organs affected. The age of the CE patients ranged from 4 to 72 years (median: 39.5, standard deviation: 14.8) (Table 2). Ten percent of the patients reported having another family member (not surveyed) also diagnosed with CE. Nearly three-quarters of the patients (74% (37/50)) originated from a rural area, 42% had only obtained a primary school level of education, and 24% had never been to school. Approximately half (54%) of the patients (87% of female patients) undertook domestic duties, with 16% of all patients working as farmers (42% of male patients), and 12% were unemployed.

Table 3 summarises the self-reported practices adopted by the patients. Of the surveyed patients, 20 (40%) owned one or more dogs. Of these, six reported allowing their dog(s) to roam freely, while five reported tying up their dog(s) within 50 m of their house. Forty percent of dog owners never allowed their dog to access the kitchen or food preparation area, and 50% never allowed their dog to access water storage containers. Sixty percent of dog owners reported feeding raw offal to their dog(s). 

Many of the patients (78%) reported the presence of a large number of dogs roaming freely about their village. Approximately half (48%) of the participants had slaughtered livestock at home, and no one had contacted a veterinarian when they had observed/detected a cyst or lesion characteristic of CE in the viscera of the slaughtered animal(s). The majority (86%) of the participants reported that they never boiled water before drinking it; however, most participants (90%) did store their water in covered containers. Additionally, a large proportion of the respondents (72%) reported receiving their water from a reverse osmosis (RO) system. Noteworthy, 26% of participants reported eating vegetables without washing. Additionally, 8% rarely washed vegetables, and 40% reported that they sometimes washed vegetables before eating them.

In Table 4 the patient’s knowledge about hydatid cysts and their attitudes toward handling potentially infected offal of slaughtered animals are outlined. A large proportion of participants (72%) had not heard about hydatid cysts before surgery. Additionally, 57% were not aware of how the disease is transmitted. A significant number of patients (70%) reported that they had not received any information from doctors or nurses about how they might become infected with CE. Furthermore, the majority (86%) indicated that they had not received any advice from medical staff on the methods to prevent reinfection by hydatid cysts. Surprisingly, half (50%) of the participants who recognized offal from slaughtered animals as unsuitable for human consumption would still consider feeding such affected offal to their dog(s). Similarly, over 81% of the respondents would consider throwing offal deemed unsuitable for human consumption into their uncovered home garbage.

## 4. Discussion

The purpose of this descriptive study was to sketch socio-demographic characteristics, household practices, and attitudes toward CE among a cohort of 50 surgically operated Iraqi patients from Basrah. Among the interviewees enrolled in this study, cysts were most commonly reported in the liver and lungs, as has been reported by others [19,20]. Added to that, the obvious representativeness of female interviewees is in line with a previous finding from a retrospective study in Iraq where we reported that CE affected more females (61.2%) than males (38.8%) [15]. Females might be more frequently exposed to the infection than males due to being engaged more with domestic activities including feeding of dogs and preparing food for the family. A higher occurrence of CE in females has similarly been reported in other countries including Jordan, Tunisia, and Iran [20,21,22]. 

In the present research, 40% of the patients affected with CE owned one or more dogs and 60% of those own dogs reported feeding them with raw offal from home slaughtered animals (typically sheep). The close association of people with dogs, combined with feeding uncooked offal, enhances the likelihood of the transmission of this zoonotic tapeworm along with environmental contamination [23,24]. A significant number of the interviewed cases (78%) reported the presence of a large number of dogs roaming freely in their village. Stray or free-roaming dogs are considered a major source of CE for humans [25]. A study in Tripoli, Libya reported that 25.8% of stray dogs had *E. granulosus*, primarily arising from access to offal and scavenging from dead animals [26]. It is recommended that the veterinary services in Basrah undertake steps to reduce the number of stray dogs which constitute a significant public health concern, not only for echinococcosis but other zoonotic diseases, such as rabies and toxocariasis [27,28]. A control programme should also consider treating domestic and stray dogs with the anthelmintic (e.g., praziquantel). In Western China, monthly dosing of stray dogs over a four year period with praziquantel resulted in a reduction in the prevalence of infected dogs from 18.6% to 0% [29]. 

Around half (52%) of the interviewees reported slaughtering animals at home for household consumption. Additionally, all of the respondents who slaughtered their animals reported that they never notify a veterinarian or meat inspector if they find cysts in the animal tissue. Other regional studies have highlighted the common practice of slaughtering animals by households in or near their homes as a risk factor for CE [10,30]. Raising community awareness about the importance of slaughtering animals in an abattoir rather than at home in the backyard, under the supervision of a veterinarian, could help in limiting the opportunity for the completion of the *Echinococcus* life cycle through appropriate disposal of affected offal [31], as well as reducing exposure of humans to other zoonotic pathogens [32]. 

Although RO purchased water was commonly used among the interviewees in this study, 86% reported they never boiled water before drinking it. The RO water in the study area is not sourced from home-installed systems, rather it is sold by whole distributers using industrial size RO units. Uncontrolled distribution, transportation, and storage conditions could serve as a window for post-treatment contamination of the water supply with CE and other pathogens. Keeping water safe and away from free-roaming dogs would also help in reducing the potential transmission of the disease, as reported by others [31,33]. Studies in Jordan [34] and Kenya [35] indicated that contaminated drinking water was a risk factor for human CE, with *Echinococcus* eggs detected in the water used by both people and livestock. Consequently, treatment of water before drinking (e.g., boiling) is an important process to minimize the risk of disease transmission, especially in areas lacking a well-controlled water supply system. 

In this study, 26% of the participants reported never washing vegetables prior to consumption. These results are similar to another study in Jordan involving 55 patients infected with CE, which revealed that, in addition to contact with animals through their occupation, many of the patients also consumed raw vegetables [36]. Eating unwashed vegetables is a risky practice potentially increasing exposure to *Echinococcus*, as well as other zoonotic diseases [37,38]. A study in Turkey identified a variety of canine parasite eggs on unwashed vegetables, including *Taenia* spp. (3.5%), *Toxocara* spp. (1.5%) and *Ascaris lumbricoides* (1.0%) [39]. Low levels of awareness regarding the risks associated with eating unwashed vegetables is considered an important factor for possibly acquiring CE in humans [40]. 

It is crucial that the general public is made aware of the risk of echinococcosis from the consumption of potentially contaminated food or water; given the fact that eggs from *Echinococcus* could survive for nearly 41 months in an arid climate under ideal environmental conditions [41], combined with the high prevalence of infection in dogs [42], and a large free-roaming dog population. The majority of participants (cases) in the current study had not heard of CE, and it is of more concern that 57% did not know about the mode of transmission even after going through a major surgery treatment to recover from such disease. This is consistent with the results of other questionnaire surveys that have been conducted in Libya and Morocco [26,43], with many of the respondents having limited to no knowledge about echinococcosis. Currently, there is no control program for echinococcosis or educational campaign in Basrah, putting the community at a disadvantage regarding this important zoonosis.

The current study found that most participants followed poor practices concerning the disposal of offal unsuitable for human consumption. Offal from slaughtered livestock that is unsuitable for human consumption needs to be disposed of by burning, burying, or rendering to break the life cycle of *Echinococcus* [44,45]. A sizable proportion of the respondents (~40%) were not aware of the risks associated with dogs and other carnivores having access to raw offal and how it can be involved in the transmission of *Echinococcus*. Collectively, these results call for the need for a One Health approach of collaboration between the Health Department of Basrah and the Veterinary Authorities to develop and implement educational programs on echinococcosis for farmers, dog owners, and the general public. Such One Health educational programs should provide information on: The importance of regular deworming of dogs; the need for improved hygiene during food preparation; going away from the habit of slaughtering animals at their homes to slaughtering it in the local abattoir; communicating strict guidelines on how to dispose of infected livestock offal; and communicating practices to minimize infection from dogs. It is also recommended to increase awareness on CE among medical professionals, who could help to disseminate advice on preventative measures to their patients.

## 5. Conclusions 

Many of the interviewed cases in this study owned dogs and reported that large numbers of free-roaming dogs were in the vicinity of where the cases lived. Added to that, small ruminants were commonly slaughtered for consumption by many of the patients at their homes. Gaps in knowledge were evident regarding washing vegetables prior to eating them; realizing how echinococcosis is transmitted to humans; and knowledge about the sanitary disposal of affected offal. It is strongly recommended that a control programme for CE is implemented to reduce the disease in humans, livestock, and dogs, and an educational campaign be developed for the general public to raise awareness about practices that could help in reducing the disease transmission and the human–animal–environment interface. Moreover, this study emphasizes the lack of preventive health information provided by the medical staff for the people who had gone through surgery treatment. In CE endemic areas, the opportunity for applying the One Health approach should always be considered in order to avoid primary infections and avoiding further reinfections. 

## Figures and Tables

**Table 1:** Anatomical sites of hydatid cysts as reported by the patients (*n* = 50).

	Female	Male	Total (%)
**Affected organs**			
Lung	10 (25.6)	7 (33.3)	17 (28.3)
Liver	18 (46.1)	13 (61.9)	31 (51.7)
Urinary bladder	1 (2.5)	1 (4.7)	2 (3.3)
Spleen	4 (10.2)	0	4 (6.7)
Kidney	2 (5.1)	0	2 (3.3)
Ovary	2 (5.1)	0	2 (3.3)
Pancreas	1 (2.5)	0	1 (1.7)
Intestine	1 (2.5)	0	1 (1.7)
Total	39 (65.0)	21 (35.0)	
**Number of organs affected per patients**			
One	24 (77.4)	17 (89.4)	41 (82.0)
Two	6 (19.3)	2 (10.5)	8 (16.0)
Three	1 (3.2)	0	1 (2.0)
Total	31 (62.0)	19 (38.0)	

**Table 2:** Descriptive characteristics of the enrolled participants (*n* = 50).

Variable	Categories	*n*	Percentage (95% CI)
Gender	Female	31	62 (47.2, 75.3)
	Male	19	38 (24.7, 55.8)
Age of patients (years)	<10	2	4 (0.5, 13.7)
	11–20	5	10 (3.3, 21.8)
	21–30	10	20 (10.0, 33.7)
	31–40	10	20 (10.0, 33.7)
	41–50	14	28 (16.2, 42.5)
	51–60	6	12 (4.5, 24.3)
	>61	3	6 (1.3, 16.5)
Has any other member in your family been diagnosed with CE?	Yes	5	10 (3.3, 21.8)
	No	45	90 (78.2, 96.7)
Residency	Rural	37	74 (59.7, 85.4)
	Urban	13	26 (14.6, 40.3)
Education level	Never went to school	12	24 (13.1, 38.2)
	Literacy class	5	10 (3.3, 21.8)
	Primary	21	42 (28.2, 56.8)
	Secondary	10	20 (10.0, 33.7)
	College	2	4 (0.5, 13.7)
Occupation	Public servant	2	4 (0.5, 13.7)
	Farmer	8	16 (7.2, 29.1)
	Housewife	27	54 (39.3, 68.2)
	Student	2	4 (0.5, 13.7)
	Unemployed	6	12 (4.5, 24.3)
	Other	5	10 (3.3, 21.8)

**Table 3:** Descriptive self-reported practices toward cystic echinococcosis.

Questions	Categories	*n*	Percentage (95% CI)
Do you own a dog (s)?	Yes	20	40 (26.4, 54.8)
	No	30	60 (45.2, 73.6)
If you own a dog is it tied up?	Yes	6	30 (11.9, 54.3)
	No	14	70 (45.7, 88.1)
If a dog is tied up, where is it tied up?	Far (>50 m) from my dwelling	1	16.7 (0.4, 64.1)
	Near (<50 m) to my dwelling	5	83.3 (53.9, 99.6)
Does your dog have access to the kitchen area/food preparing area?	Never	8	40 (19.1, 63.9)
	Rarely	2	10 (1.2, 31.7)
	Sometimes	6	30 (11.9, 54.3)
	Often	2	10 (1.2, 31.7)
	Always	2	10 (1.2, 31.7)
Does your dog have access to containers used to store drinking water for people?	Never	10	50 (27.2, 72.8)
	Rarely	2	10 (1.2, 31.7)
	Sometimes	7	35 (15.4, 59.2)
	Often	1	5.0 (0.1, 24.9)
	Always	0	0.0 (0.9, 16.8)
How often do you feed your dog raw offal (for example liver or lung)?	Never	8	40 (19.1, 63.9)
	Rarely	1	5 (0.1, 24.9)
	Sometimes	5	25 (8.7, 49.1)
	Often	4	20 (5.7, 43.7)
	Always	2	10 (1.2, 31.7)
Have you seen stray dogs in your neighborhood over the last week?	Yes	39	78 (64.0, 88.5)
	No	11	22 (11.5, 36.0)
Do you own livestock?	Yes	22	44 (30.0, 58.7)
	No	28	56 (41.3, 70.0)
Do you slaughter livestock at your home?	Yes	24	48 (33.7, 62.6)
	No	26	52 (37.4, 66.3)
Do you report to an inspector (vet or meat inspector) if there is a cyst in the slaughtered carcass?	Yes	0	0.0 (0.0, 14.3)
	No	24	48 (27.4, 69.1)
What is the main source of family drinking water?	Reverse Osmosis (RO)	36	72 (57.5, 83.8)
	Tanker	9	18 (8.6, 31.4)
	Tap water	4	8 (2.2, 19.2)
	Well	1	2 (0.1, 10.6)
Do you boil water before drinking it?	Never	43	86 (73.3, 94.2)
	Some of the time	4	8 (2.2, 19.2)
	Most of the time	2	4 (0.5, 13.7)
	All the time	1	2 (0.1, 10.6)
How is your family’s drinking water stored?	Stored in covered containers	45	90 (78.2, 96.7)
	Stored in uncovered containers	5	10 (3.3, 21.8)
How often do you eat leafy vegetables without washing them?	Never	13	26 (14.6, 40.3)
	Rarely	4	8 (2.2, 19.2)
	Sometimes	20	40 (26.4, 54.8)
	Often	4	8 (2.2, 19.2)
	Always	9	18 (8.6, 31.4)
For lettuce and other leafy vegetables, how would you prepare them as part of your salad?	Wash it under running water only	36	72 (57.5, 83.8)
	Soak in water in the sink	10	20 (10.0, 33.7)
	Wash it with detergent	3	6 (1.3, 16.5)
	Peel outer leaves & eat the rest	1	2 (0.1, 10.6)

**Table 4:** Descriptive self-reported knowledge and attitudes toward cystic echinococcosis.

Variables	Categories	*n*	Percentage (95% CI)
Before getting sick, had you heard about cystic echinococcosis or hydatid cyst disease?	Yes	14	28 (16.2, 42.5)
	No	36	72 (57, 5, 83.8)
If you answered yes in the previous question (*n* = 14), what do you think could be the source of the infection you have contracted?	Not sure	8	57 (28.9, 82.3)
	Dog	1	7.14 (0.2, 33.9)
	Food	4	28.57 (8.4, 58.1)
	Water	1	7.14 (0.2, 33.9)
Did your doctor, nurse or other medical staff explain how you became infected with a hydatid cyst?	Yes	15	30 (17.9, 44.9)
	No	35	70 (55.4, 82.1)
Did your doctor, nurse or other medical staff explain ways of how to protect you from future infection?	Yes	7	14 (5.8, 26.7)
	No	43	86 (73.3, 94.2)
Would you feed animal’s offal that is not suitable for human consumption to your dog? *	Would definitely consider doing it	10	50 (27.2, 72.8)
	Might consider doing it	7	35 (15.4, 59.2)
	Would definitely not do it	3	15 (3.2, 37.9)
Would you throw offal that is not suitable for human consumption into a communal open rubbish area? **	Would definitely consider doing it	13	54.17 (32.8, 74.4)
	Might consider doing it	9	37.50 (18.8, 59.4)
	Would definitely not do it	2	8.33 (1.0, 27.0)
Would you burn to ash offal that is not suitable for human consumption? **	Would definitely consider doing it	1	4.17 (0.1, 21.1)
	Might consider doing it	9	37.50 (18.8, 59.4)
	Would definitely not do it	14	58.33 (36.6, 77.9)
Would you bury organs not suitable for human consumption? **	Would definitely consider doing it	2	8.33 (1.0, 27.0)
	Might consider doing it	8	33.33 (15.6, 55.3)
	Would definitely not do it	14	58.33 (36.6, 77.9)

* Answers from only those who owned dogs (*n* = 20); ** for offal disposal, one or more categories were allowed.

## References

[1] Deplazes P., Rinaldi L., Alvarez Rojas C.A., Torgerson P.R., Harandi M.F., Romig T., Antolova D., Schurer J.M., Lahmar S., Cringoli G. (2017). Global Distribution of Alveolar and Cystic Echinococcosis. Adv. Parasitol..

[2] Romig T., Deplazes P., Jenkins D., Giraudoux P., Massolo A., Craig P.S., Wassermann M., Takahashi K., de la Rue M. (2017). Ecology and Life Cycle Patterns of *Echinococcus* Species. Adv. Parasitol..

[3] Acha P.N., Szyfres B. (1987). Parasite Zoonosis: Helminthiases. Zoonoses and Communicable Diseases Common to Man and Animals.

[4] Torgerson P.R., Budke C.M. (2003). Echinococcosis—An international public health challenge. Res. Vet. Sci..

[5] Eckert J., Deplazes P. (2004). Biological, Epidemiological, and Clinical Aspects of Echinococcosis, a Zoonosis of Increasing Concern. Clin. Microbiol. Rev..

[6] Craig P.S., McManus D.P., Lightowlers M.W., Chabalgoity J.A., Garcia H.H., Gavidia C.M., Gilman R.H., Gonzalez A.E., Lorca M. (2007). Prevention and control of cystic Echinococcosis. Lancet Infect. Dis..

[7] Moro P., Schantz P.M. (2009). Echinococcosis: A review. Int. J. Infect. Dis..

[8] Heidari Z., Mohebali M., Zarei Z., Aryayipour M., Eshraghian M.R., Kia E.B., Shodajei S., Abdi J., Rakhshanpour A., Rokni M. (2011). Seroepidemiological Study of Human Hydatidosis in Meshkinshahr District, Ardabil Province, Iran. Iran J. Parasitol..

[9] Zhang T., Zhao W., Yang D., Piao D., Huang S., Mi Y., Zhao X., Cao J., Shen Y., Zhang W., Liu A. (2015). Human cystic echinococcosis in Heilongjiang Province, China: A retrospective study. BMC Gastroenterol..

[10] Galeh T.M., Spotin A., Mahami-Oskouei M., Carmena D., Rahimi M.T., Barac A., Ghoyounchi R., Berahmat R., Ahmadpour E. (2018). The seroprevalence rate and population genetic structure of human cystic echinococcosis in the Middle East: A systematic review and meta-analysis. Int. J. Surg..

[11] CDC (2012). Communicable Diseases Control Guidelines.

[12] Maktoof A.R., Abu Tabeekh M.A.S. (2015). Classification of Endemicity of Cystic Echinococcosis in Basra Governorate-Iraq. SJAR.

[13] Thweni M.M., Yassen L.J. (2015). Hepatic Hydatidosis in man and livestock in Nassiriyah, Iraq. Int. J. PharmTech Res..

[14] Al-Yasari H.F., Al-Shaiely A.K. (2013). A Study of Human Hydatidosis: Demographically and Clinically In Hilla City. J. Pure Appl. Sci..

[15] Abdulhameed M.F., Habib I., Al-Azizz S.A., Robertson I. (2018). A retrospective study of human cystic echinococcosis in Basrah province, Iraq. Acta Trop..

[16] Kern P., Menezes da Silva A., Akhan O., Mullhaupt B., Vizcaychipi K.A., Budke C., Vuitton D.A. (2017). The Echinococcoses: Diagnosis, Clinical Management and Burden of Disease. Adv. Parasitol..

[17] NGO, Coordination Committee for Iraq Basrah Governorate Profile. http://www.ncciraq.org/images/infobygov/NCCI_Basra_Governorate_Profile.pdf.

[18] Abdulhameed M.F., Habib I., Al-Azizz S.A., Robertson I. (2018). Knowledge, Awareness and Practices Regarding Cystic Echinococcosis among Livestock Farmers in Basrah Province, Iraq. Vet. Sci..

[19] Kebede W., Hagos A., Girma Z., Lobago F. (2009). Echinococcosis/hydatidosis: Its prevalence, economic and public health significance in Tigray region, North Ethiopia. Trop. Anim. Health Prod..

[20] Al-Qaoud K.M., Craig P.S., Abdel-Hafez S.K. (2003). Retrospective surgical incidence and case distribution of cystic echinococcosis in Jordan between 1994 and 2000. Acta Trop..

[21] Lahmar S., Rebaï W., Boufana B.S., Craig P.S., Ksantini R., Daghfous A., Chebbi F., Fteriche F., Bedioui H., Jouini M. (2009). Cystic echinococcosis in Tunisia: Analysis of hydatid cysts that have been surgically removed from patients. Ann. Trop. Med. Parasitol..

[22] Hajipirloo H.M., Bozorgomid A., Alinia T., Tappeh K.H., Mahmodlou R. (2013). Human cystic echinococcosis in west Azerbaijan, northwest Iran: A retrospective hospital based survey from 2000 To 2009. Iran J. Parasitol..

[23] Schantz P.M., Wang H., Qiu J., Liu F.J., Saito E., Emshoff A., Ito A., Roberts J.M., Delker C. (2003). Echinococcosis on the Tibetan Plateau: Prevalence and risk factors for cystic and alveolar echinococcosis in Tibetan populations in Qinghai Province, China. Parasitology.

[24] Possenti A., Manzano-Román R., Sánchez-Ovejero C., Boufana B., La Torre G., Siles-Lucas M., Boufana B., La Torre G., Siles-Lucas M., Casulli A. (2016). Potential Risk Factors Associated with Human Cystic Echinococcosis: Systematic Review and Meta-analysis. PLoS Negl. Trop. Dis..

[25] Kayouèche F., Chassagne M., Benmakhlouf A., Abrial D., Dorr N., Benlatreche C., Barnouin J. (2009). Socio-ecological factors associated with risk of family hydatidosis in the wilaya of Constantine (Algeria) through interviews of urban and rural households. Rev. Med. Vet..

[26] Buishia I.E., Njorogeb E.M., Bouamrac O., Craiga P.S. (2005). Canine echinococcosis in northwest Libya: Assessment of coproantigen ELISA, and a survey of infection with analysis of risk-factors. Vet. Parasitol..

[27] Kilic B., Unal B., Semin S., Konakci S.K. (2006). An important public health problem: Rabies suspected bites and post-exposure prophylaxis in a health district in Turkey. Int. J. Infect. Dis..

[28] Macpherson C.N. (2013). The epidemiology and public health importance of toxocariasis: A zoonosis of global importance. Int. J. Parasitol..

[29] Zhang W., Zhang Z., Yimit T., Shi B., Aili H., Tulson G., You H., Li J., Gray D.J., McManus D.P. (2009). A pilot study for control of hyperendemic cystic hydatid disease in China. PLoS Negl. Trop. Dis..

[30] Nasrieh M.A., Abdel-Hafez S.K., Kamhawi S.A., Craig P.S., Schantz P.M. (2003). Cystic echinococcosis in Jordan: Socioeconomic evaluation and risk factors. Parasitol. Res..

[31] Ito A., Urbani C., Jiamin Q., Vuitton D.A., Dongchuan Q., Heath D.D., Craig P.S., Zheng F., Schantz P.M. (2003). Control of echinococcosis and cysticercosis: A public health challenge to international cooperation in China. Acta Trop..

[32] Jimenez S., Perez A., Gil H., Schantz P.M., Ramalle E., Juste R.A. (2002). Progress in control of cystic echinococcosis in La Rioja, Spain: Decline in infection prevalences in human and animal hosts and economic costs and benefits. Acta Trop..

[33] Moro P.L., Cavero C.A., Tambini M., Briceno Y., Jimenez R., Cabrera L. (2008). Identification of risk factors for cystic echinococcosis in a peri-urban population of Peru. Trans. R. Soc. Trop. Med. Hyg..

[34] Dowling P.M., Abo-Shehada M.N., Torgerson P.R. (2000). Risk factors associated with human cystic echinococcosis in Jordan: Results of a case-control study. Ann. Trop. Med. Parasitol..

[35] Craig P.S., Macpherson C.N., Watson-Jones D.L., Nelson G.S. (1988). Immunodetection of *Echinococcus* eggs from naturally infected dogs and from environmental contamination sites in settlements in Turkana, Kenya. Trans. R. Soc. Trop. Med. Hyg..

[36] Yaghan R.J., Bani-Hani K.E., Heis H.A. (2004). The clinical and epidemiological features of hydatid disease in Northern Jordan. Saudi Med. J..

[37] Qaqish A.M., Nasrieh M.A., Al-Qaoud K.M., Craig P.S., Abdel-Hafez S.K. (2003). The seroprevalences of cystic echinococcosis, and the associated risk factors, in rural-agricultural, bedouin and semi-bedouin communities in Jordan. Ann. Trop. Med. Parasitol..

[38] Federer K., Armua-Fernandez M.T., Gori F., Hoby S., Wenker C., Deplazes P. (2016). Detection of taeniid (*Taenia* spp., *Echinococcus* spp.) eggs contaminating vegetables and fruits sold in European markets and the risk for metacestode infections in captive primates. Int. J. Parasitol. Parasites Wildl..

[39] Kozan E., Gonenc B., Sarimehmetoglu O., Aycicek H. (2005). Prevalence of helminth eggs on raw vegetables used for salads. Food Control.

[40] Aydın M.F., Adıgüzel E., Güzel H. (2018). A study to assess the awareness of risk factors of cystic echinococcosis in Turkey. Saudi Med. J..

[41] Thevenet P.S., Jensen O., Drut R., Cerrone G.E., Grenovero M.S., Alvarez H.M., Targovnik H.M., Basualdo J.A. (2005). Viability and infectiousness of eggs of *Echinococcus granulosus* aged under natural conditions of inferior arid climate. Vet. Parasitol..

[42] Beiromvand M., Akhlaghi L., Massom S.H.F., Meamar A.R., Motevalian A., Oormazdi H., Razmjou E. (2013). Prevalence of zonnotic intestinal parasites in domestic and stray dogs in a rural area of Iran. Prev. Vet. Med..

[43] El Berbri I., Ducrotoy M.J., Petavy A., Fassifihri O., Shaw A.P., Bouslikhane M., Boue F., Welburn S.C., Dakkak A. (2015). Knowledge, attitudes and practices with regard to the presence, transmission, impact, and control of cystic echinococcosis in Sidi Kacem Province, Morocco. Infect. Dis. Pov..

[44] Acosta-Jamett G., Cleaveland S., Bronsvoort B.M., Cunningham A.A., Bradshaw H., Craig P.S. (2010). *Echinococcus**granulosus* infection in domestic dogs in urban and rural areas of the Coquimbo region, north-central Chile. Vet. Parasitol..

[45] Wachira T.M., Macpherson C.N.L., Gathuma J.M. (1991). Release and survival of *Echinococcus* eggs in different environments in Turkana, and their possible impact on the incidence of hydatidosis in man and livestock. J. Helminthol..

